# 7-Eth­oxy-4′-methoxy­isoflavone (monoethyl­formononetin)

**DOI:** 10.1107/S1600536808010672

**Published:** 2008-04-23

**Authors:** Qiu-Ya Wang

**Affiliations:** a Department of Chemistry and Chemical Engineering, Weinan Teachers University, weinan 714000, People’s Republic of China

## Abstract

The title compound, C_18_H_16_O_4_, is composed of a benzopyran­one core with a 4-methoxy­phenyl subsituent in the 3-position and an additional eth­oxy group in the 7-position. The benzopyran­one ring is not coplanar with the benzene ring, the dihedral angle between them being 41.76 (7)°. The meth­oxy and eth­oxy substituents are nearly coplanar with the ring systems to which they are attached. Individual mol­ecules are linked by two kinds of inter­molecular hydrogen bonds into chains containing classical *R*_2_^2^(8) rings. The chains are further assembled by aromatic F-tape and T-tape stacking inter­actions and additional inter­molecular hydrogen bonding to give a two-dimensional network.

## Related literature

For related literature, see: Cassidy *et al.* (1994 ▶); Janiak (2000 ▶); Jha *et al.* (1985 ▶); Potter (1995 ▶); Sirtori *et al.* (1995 ▶); Zhang *et al.* (2005 ▶).
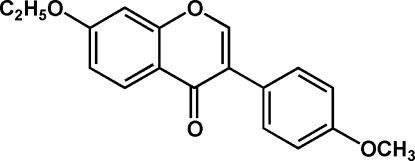


## Experimental

### 

#### Crystal data

C_18_H_16_O_4_
*M* *_r_* = 296.31
Triclinic, 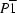

*a* = 6.234 (3) Å
*b* = 10.257 (5) Å
*c* = 12.159 (6) Å
α = 80.837 (7)°
β = 87.728 (8)°
γ = 73.653 (7)°
*V* = 736.5 (6) Å^3^
*Z* = 2
Mo *K*α radiation
μ = 0.09 mm^−1^
*T* = 296 (2) K
0.35 × 0.31 × 0.27 mm

#### Data collection

Bruker SMART CCD area-detector diffractometer
Absorption correction: multi-scan (*SADABS*; Bruker, 1999 ▶) *T*_min_ = 0.968, *T*_max_ = 0.975
3678 measured reflections
2572 independent reflections
1732 reflections with *I* > 2σ(*I*)
*R*_int_ = 0.019

#### Refinement

*R*[*F* ^2^ > 2σ(*F* ^2^)] = 0.040
*wR*(*F* ^2^) = 0.123
*S* = 1.04
2572 reflections
202 parameters
H-atom parameters constrained
Δρ_max_ = 0.18 e Å^−3^
Δρ_min_ = −0.15 e Å^−3^

### 

Data collection: *SMART* (Bruker, 1999 ▶); cell refinement: *SAINT-Plus* (Bruker, 1999 ▶); data reduction: *SAINT-Plus*; program(s) used to solve structure: *SHELXS97* (Sheldrick, 2008 ▶); program(s) used to refine structure: *SHELXL97* (Sheldrick, 2008 ▶); molecular graphics: *SHELXTL* (Sheldrick, 2008 ▶); software used to prepare material for publication: *SHELXTL*.

## Supplementary Material

Crystal structure: contains datablocks I, global. DOI: 10.1107/S1600536808010672/im2059sup1.cif

Structure factors: contains datablocks I. DOI: 10.1107/S1600536808010672/im2059Isup2.hkl

Additional supplementary materials:  crystallographic information; 3D view; checkCIF report

## Figures and Tables

**Table 1:** Hydrogen-bond geometry (Å, °)

*D*—H⋯*A*	*D*—H	H⋯*A*	*D*⋯*A*	*D*—H⋯*A*
C9—H9⋯O2^i^	0.93	2.52	3.105 (2)	121
C16—H16⋯O3^ii^	0.93	2.60	3.315 (2)	134
C17—H17*B*⋯O2^iii^	0.97	2.58	3.358 (3)	138

Symmetry codes: (i) 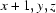; (ii) 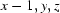; (iii) 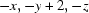.

**Fig. 1.**
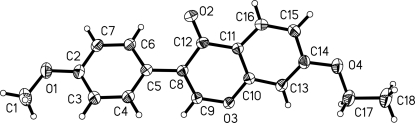
The molecular structure of (I), showing the atom-numbering scheme and 30% probability displacement ellipsoids.

**Fig. 2.**
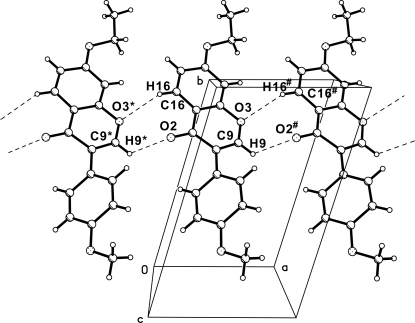
The one-dimensional chain formed via two intermolecular hydrogen bonds of the C–H···O type. Atoms marked with an asterisk (*) or a hash (#) are at the symmetry positions (-x + 1, y, z) and (x + 1, y, z), respectively.

**Fig. 3.**
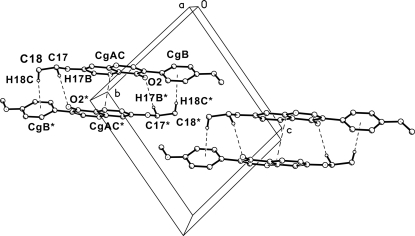
The dimeric structure formed via aromatic F-tape and T-tape stacking interactions as well as another intermolecular hydrogen bond of the C–H···O type. Atoms marked with an asterisk (*) are at the symmetry positions (-x, 2 - y, -z). For the sake of clarity, some H atoms of isoflavone skeletons have been omitted.

## supplementary crystallographic information

### Comment

The isoflavone derivative formononetin (4'-methoxy-7-hydroxyisoflavone) displays a wide range of biological activities, such as antioxidant effect (Jha *et al.*, 1985), inhibiting cardiovascular disease (Potter *et al.*, 1995; Sirtori *et al.*, 1995) and balancing women's hormones (Cassidy *et al.*, 1994). The title compound 4'-methoxy-7-ethoxylisoflavone, (I), is a derivative of formononetin and has potential medical applications.

The title compound is composed of a benzopyranone core with a *p*-methoxyphenyl substituent in 3-position and an additional ethoxy group in 7-position (Fig. 1). The geometry of the isoflavone skeleton of (I) is similar to that of monoethyldaidzein (Zhang *et al.*, 2005) with respect to most of the bond distances and angles. The atoms of the benzopyranone moiety, including ring A (C10,C11,C13–C16) and C (O3/C8—C12), display an almost coplanar configuration with a mean deviation to the least square plane of 0.0152 (17) Å. To minimize steric pressure, the two rigid ring systems, benzene ring B (C2—C7) and the benzopyranone moiety, are rotated by 41.76 (7)° with respect to each other. The methoxy group at atom C2 is nearly coplanar with the benzopyranone moiety, as indicated by the torsional angle C1—O1—C2—C3 = 4.6 (3)°; The ethoxy group at atom C14 is also nearly coplanar with the attached ring, the torsional angle C17—O4—C14—C13 being -7.9 (2)°.

A one-dimensional infinite chain is formed by two intermolecular hydrogen bonds of the C–H···O type (C9—H9···O2 and C16—H16···O3, Fig. 2, Table 1). This combination of hydrogen bonds generates *R*_2_^2^(8) rings propagating as an infinite one-dimensional chain along *a* axis.

As shown in Fig. 3, two isoflavone skeletons arrange in an anti-parallel fashion with aromatic F-tape and T-tape stacking interactions linking two molecules into a dimeric structure. The two benzopyranone moieties stack with each other with a CgAC-CgAC* distance of 3.686 (2) Å (CgAC and CgAC* are the centres of benzopyranone moieties at (*x*, *y*, *z*) and (-*x*, 2 - *y*, -*z*), respectively). The interplanar spacing measures to 3.425 (2) Å. In addition, another T-tape stacking interaction between H18C and CgB* (the centroid of ring B at (-*x*, 2 - *y*, -*z*)) of 2.818 (3) Å is observed. Both distances obviously lie in the normal range of F-tape and T-tape π-π stacking interactions (Janiak, 2000). At the same time, the carbonyl oxygen atom O2 acts as a hydrogen bond acceptor towards H17B of a neighboring molecule also leading to the formation of a dimeric substructure. The intermolecular hydrogen bond C17—H17B···O2 together with the aromatic F-tape and T-tape stacking interactions assemble the chains mentioned above into a two-dimensional network structure.

### Experimental

Formononetin (1.0 g) was dissolved into acetone (30 ml) and KOH (1 ml, 0.3%). Diethylsulfate (1 ml) was added dropwise to the solution under vigorous stirring. The mixture was stirred at room temperature for 3 h and then poured into water (50 ml). A white precipitation appeared, which was filtered after 4 h and terated with NaOH (50 ml, 2 mol/*L*) to remove traces of remaining formonetin. The precipitate was filtered and washed with water until the pH of the filtrate was 7 yielding the title compound, 4'-methoxy-7-ethoxylisoflavone (yield: 82.4%). Crystals of (I) suitable for X-ray analysis were obtained by slow evaporation of the solvent from an ethanolic solution after 3 d at room temperature.

### Refinement

H atoms bound to O atoms were found in difference maps and refined using a riding model. H atoms bound to C atoms were placed in calculated positions (C—H=0.93–0.97 Å) and refined using a riding model, allowing for free rotation of the rigid methyl groups. *U*_iso_(H) values were constrained to be 1.2Ueq (attached atom) [1.5Ueq(C) for methyl H atoms].

### Crystal data

**Table:**

C_18_H_16_O_4_	*V* = 736.5 (6) Å^3^
*M**_r_* = 296.31	*Z* = 2
Triclinic, *P*1	*F*_000_ = 312
Hall symbol: -P 1	*D*_x_ = 1.336 Mg m^−^^3^
*a* = 6.234 (3) Å	Mo *K*α radiation λ = 0.71073 Å
*b* = 10.257 (5) Å	Cell parameters from 2633 reflections
*c* = 12.159 (6) Å	µ = 0.09 mm^−^^1^
α = 80.837 (7)º	*T* = 296 (2) K
β = 87.728 (8)º	Hexagonal, colorless
γ = 73.653 (7)º	0.35 × 0.31 × 0.27 mm

### Data collection

**Table:**

Bruker SMART CCD area-detector diffractometer	2572 independent reflections
Radiation source: fine-focus sealed tube	1732 reflections with *I* > 2σ(*I*)
Monochromator: graphite	*R*_int_ = 0.019
*T* = 296(2) K	θ_max_ = 25.1º
φ and ω scans	*h* = −6→7
Absorption correction: multi-scan(SADABS; Bruker, 1999)	*k* = −12→11
*T*_min_ = 0.968, *T*_max_ = 0.975	*l* = −13→14
3678 measured reflections	

### Refinement

**Table:**

Refinement on *F*^2^	Hydrogen site location: inferred from neighbouring sites
Least-squares matrix: full	H-atom parameters constrained
*R*[*F*^2^ > 2σ(*F*^2^)] = 0.040	*w* = 1/[σ^2^(*F*_o_^2^) + (0.0722*P*)^2^] where *P* = (*F*_o_^2^ + 2*F*_c_^2^)/3
*wR*(*F*^2^) = 0.123	(Δ/σ)_max_ < 0.001
*S* = 1.04	Δρ_max_ = 0.18 e Å^−^^3^
2572 reflections	Δρ_min_ = −0.15 e Å^−^^3^
202 parameters	Extinction correction: SHELXL97 (Sheldrick, 2008), Fc^*^=kFc[1+0.001xFc^2^λ^3^/sin(2θ)]^-1/4^
Primary atom site location: structure-invariant direct methods	Extinction coefficient: 0.021 (5)
Secondary atom site location: difference Fourier map	

### Special details

**Table:**

Geometry. All e.s.d.'s (except the e.s.d. in the dihedral angle between two l.s. planes) are estimated using the full covariance matrix. The cell e.s.d.'s are taken into account individually in the estimation of e.s.d.'s in distances, angles and torsion angles; correlations between e.s.d.'s in cell parameters are only used when they are defined by crystal symmetry. An approximate (isotropic) treatment of cell e.s.d.'s is used for estimating e.s.d.'s involving l.s. planes.
Refinement. Refinement of *F*^2^ against ALL reflections. The weighted *R*-factor *wR* and goodness of fit *S* are based on *F*^2^, conventional *R*-factors *R* are based on *F*, with *F* set to zero for negative *F*^2^. The threshold expression of *F*^2^ > σ(*F*^2^) is used only for calculating *R*-factors(gt) *etc*. and is not relevant to the choice of reflections for refinement. *R*-factors based on *F*^2^ are statistically about twice as large as those based on *F*, and *R*- factors based on ALL data will be even larger.

### Fractional atomic coordinates and isotropic or equivalent isotropic displacement parameters (Å^2^)

**Table:**

	*x*	*y*	*z*	*U*_iso_*/*U*_eq_	
O1	0.4540 (2)	0.17252 (13)	0.40797 (10)	0.0661 (4)	
O2	−0.17446 (17)	0.70638 (13)	0.09853 (9)	0.0568 (4)	
O3	0.36722 (16)	0.77566 (12)	−0.07427 (9)	0.0501 (3)	
O4	−0.0813 (2)	1.17079 (13)	−0.30131 (10)	0.0628 (4)	
C1	0.6401 (4)	0.1505 (2)	0.48100 (17)	0.0836 (7)	
H1A	0.7738	0.1451	0.4379	0.125*	
H1B	0.6568	0.0660	0.5314	0.125*	
H1C	0.6143	0.2254	0.5227	0.125*	
C2	0.4019 (3)	0.29134 (18)	0.33118 (13)	0.0518 (5)	
C3	0.5249 (3)	0.3858 (2)	0.31458 (14)	0.0574 (5)	
H3	0.6505	0.3730	0.3583	0.069*	
C4	0.4600 (3)	0.49962 (18)	0.23260 (13)	0.0518 (5)	
H4	0.5444	0.5622	0.2219	0.062*	
C5	0.2730 (3)	0.52354 (17)	0.16568 (13)	0.0432 (4)	
C6	0.1484 (3)	0.42862 (17)	0.18588 (13)	0.0459 (4)	
H6	0.0202	0.4425	0.1439	0.055*	
C7	0.2120 (3)	0.31489 (18)	0.26689 (13)	0.0495 (4)	
H7	0.1264	0.2529	0.2787	0.059*	
C8	0.2177 (2)	0.64102 (16)	0.07294 (12)	0.0403 (4)	
C9	0.3870 (3)	0.67076 (17)	0.01147 (13)	0.0459 (4)	
H9	0.5303	0.6143	0.0294	0.055*	
C10	0.1585 (2)	0.85994 (16)	−0.10328 (12)	0.0413 (4)	
C11	−0.0298 (2)	0.83924 (16)	−0.04665 (12)	0.0394 (4)	
C12	−0.0106 (2)	0.72643 (17)	0.04624 (12)	0.0417 (4)	
C13	0.1508 (3)	0.96676 (18)	−0.19021 (13)	0.0480 (4)	
H13	0.2807	0.9761	−0.2269	0.058*	
C14	−0.0519 (3)	1.05814 (17)	−0.22079 (13)	0.0476 (4)	
C15	−0.2471 (3)	1.04083 (18)	−0.16642 (14)	0.0521 (5)	
H15	−0.3851	1.1025	−0.1875	0.063*	
C16	−0.2340 (3)	0.93317 (18)	−0.08245 (14)	0.0488 (4)	
H16	−0.3648	0.9219	−0.0480	0.059*	
C17	0.1156 (3)	1.2050 (2)	−0.34750 (15)	0.0656 (5)	
H17A	0.1971	1.1364	−0.3918	0.079*	
H17B	0.2131	1.2079	−0.2882	0.079*	
C18	0.0418 (4)	1.3431 (2)	−0.41900 (17)	0.0805 (6)	
H18A	−0.0447	1.3373	−0.4807	0.121*	
H18B	0.1706	1.3717	−0.4463	0.121*	
H18C	−0.0479	1.4088	−0.3757	0.121*	

### Atomic displacement parameters (Å^2^)

**Table:**

	*U*^11^	*U*^22^	*U*^33^	*U*^12^	*U*^13^	*U*^23^
O1	0.0788 (9)	0.0575 (9)	0.0569 (8)	−0.0217 (7)	−0.0114 (6)	0.0143 (7)
O2	0.0417 (6)	0.0653 (9)	0.0612 (7)	−0.0198 (6)	0.0080 (5)	0.0033 (6)
O3	0.0385 (6)	0.0487 (8)	0.0558 (7)	−0.0100 (5)	0.0055 (5)	0.0082 (6)
O4	0.0693 (8)	0.0575 (9)	0.0555 (8)	−0.0191 (7)	−0.0097 (6)	0.0146 (7)
C1	0.0906 (16)	0.0762 (16)	0.0733 (14)	−0.0212 (12)	−0.0252 (11)	0.0223 (12)
C2	0.0603 (11)	0.0489 (11)	0.0432 (10)	−0.0163 (9)	0.0018 (8)	0.0030 (8)
C3	0.0548 (11)	0.0615 (13)	0.0542 (11)	−0.0196 (9)	−0.0114 (8)	0.0049 (9)
C4	0.0522 (10)	0.0521 (11)	0.0537 (10)	−0.0233 (9)	−0.0052 (8)	0.0020 (9)
C5	0.0418 (9)	0.0451 (10)	0.0433 (9)	−0.0149 (7)	0.0017 (7)	−0.0036 (8)
C6	0.0458 (9)	0.0490 (11)	0.0444 (9)	−0.0171 (8)	0.0007 (7)	−0.0045 (8)
C7	0.0540 (10)	0.0490 (11)	0.0486 (10)	−0.0224 (8)	0.0041 (7)	−0.0034 (8)
C8	0.0405 (8)	0.0411 (10)	0.0409 (8)	−0.0158 (7)	−0.0009 (6)	−0.0034 (7)
C9	0.0392 (9)	0.0413 (10)	0.0524 (10)	−0.0088 (7)	−0.0007 (7)	0.0029 (8)
C10	0.0398 (8)	0.0418 (10)	0.0410 (9)	−0.0107 (7)	−0.0005 (6)	−0.0036 (8)
C11	0.0387 (8)	0.0400 (10)	0.0397 (8)	−0.0126 (7)	−0.0012 (6)	−0.0037 (7)
C12	0.0401 (9)	0.0460 (10)	0.0426 (9)	−0.0174 (7)	0.0009 (7)	−0.0079 (8)
C13	0.0492 (10)	0.0486 (11)	0.0455 (10)	−0.0167 (8)	0.0046 (7)	−0.0007 (8)
C14	0.0568 (10)	0.0455 (11)	0.0397 (9)	−0.0159 (8)	−0.0069 (7)	0.0001 (8)
C15	0.0439 (10)	0.0488 (11)	0.0606 (11)	−0.0107 (8)	−0.0107 (8)	−0.0007 (9)
C16	0.0387 (9)	0.0521 (11)	0.0556 (10)	−0.0153 (8)	−0.0010 (7)	−0.0039 (9)
C17	0.0787 (13)	0.0561 (13)	0.0550 (11)	−0.0167 (10)	0.0116 (9)	0.0053 (10)
C18	0.1102 (17)	0.0565 (14)	0.0655 (13)	−0.0205 (12)	0.0130 (12)	0.0099 (11)

### Geometric parameters (Å, °)

**Table:**

O1—C2	1.379 (2)	C7—H7	0.9300
O1—C1	1.433 (2)	C8—C9	1.346 (2)
O2—C12	1.2307 (18)	C8—C12	1.464 (2)
O3—C9	1.3557 (19)	C9—H9	0.9300
O3—C10	1.3675 (18)	C10—C13	1.388 (2)
O4—C14	1.365 (2)	C10—C11	1.388 (2)
O4—C17	1.439 (2)	C11—C16	1.399 (2)
C1—H1A	0.9600	C11—C12	1.465 (2)
C1—H1B	0.9600	C13—C14	1.369 (2)
C1—H1C	0.9600	C13—H13	0.9300
C2—C3	1.383 (3)	C14—C15	1.404 (2)
C2—C7	1.387 (2)	C15—C16	1.366 (2)
C3—C4	1.384 (2)	C15—H15	0.9300
C3—H3	0.9300	C16—H16	0.9300
C4—C5	1.390 (2)	C17—C18	1.499 (3)
C4—H4	0.9300	C17—H17A	0.9700
C5—C6	1.395 (2)	C17—H17B	0.9700
C5—C8	1.485 (2)	C18—H18A	0.9600
C6—C7	1.376 (2)	C18—H18B	0.9600
C6—H6	0.9300	C18—H18C	0.9600
			
C2—O1—C1	117.47 (14)	O3—C10—C13	115.53 (13)
C9—O3—C10	118.54 (12)	O3—C10—C11	121.09 (14)
C14—O4—C17	117.60 (14)	C13—C10—C11	123.36 (15)
O1—C1—H1A	109.5	C10—C11—C16	116.07 (15)
O1—C1—H1B	109.5	C10—C11—C12	120.89 (14)
H1A—C1—H1B	109.5	C16—C11—C12	123.03 (13)
O1—C1—H1C	109.5	O2—C12—C8	122.74 (15)
H1A—C1—H1C	109.5	O2—C12—C11	122.28 (15)
H1B—C1—H1C	109.5	C8—C12—C11	114.98 (12)
O1—C2—C3	124.71 (16)	C14—C13—C10	118.61 (15)
O1—C2—C7	116.29 (15)	C14—C13—H13	120.7
C3—C2—C7	118.99 (17)	C10—C13—H13	120.7
C2—C3—C4	119.61 (16)	O4—C14—C13	124.46 (15)
C2—C3—H3	120.2	O4—C14—C15	115.48 (16)
C4—C3—H3	120.2	C13—C14—C15	120.05 (16)
C3—C4—C5	122.27 (15)	C16—C15—C14	119.81 (16)
C3—C4—H4	118.9	C16—C15—H15	120.1
C5—C4—H4	118.9	C14—C15—H15	120.1
C4—C5—C6	117.05 (15)	C15—C16—C11	122.07 (14)
C4—C5—C8	120.86 (13)	C15—C16—H16	119.0
C6—C5—C8	122.00 (14)	C11—C16—H16	119.0
C7—C6—C5	121.12 (15)	O4—C17—C18	107.74 (16)
C7—C6—H6	119.4	O4—C17—H17A	110.2
C5—C6—H6	119.4	C18—C17—H17A	110.2
C6—C7—C2	120.92 (15)	O4—C17—H17B	110.2
C6—C7—H7	119.5	C18—C17—H17B	110.2
C2—C7—H7	119.5	H17A—C17—H17B	108.5
C9—C8—C12	118.69 (15)	C17—C18—H18A	109.5
C9—C8—C5	118.03 (14)	C17—C18—H18B	109.5
C12—C8—C5	123.28 (12)	H18A—C18—H18B	109.5
C8—C9—O3	125.81 (15)	C17—C18—H18C	109.5
C8—C9—H9	117.1	H18A—C18—H18C	109.5
O3—C9—H9	117.1	H18B—C18—H18C	109.5
			
C1—O1—C2—C3	4.6 (3)	O3—C10—C11—C12	0.1 (2)
C1—O1—C2—C7	−175.63 (15)	C13—C10—C11—C12	−178.56 (13)
O1—C2—C3—C4	178.07 (15)	C9—C8—C12—O2	−179.79 (15)
C7—C2—C3—C4	−1.7 (3)	C5—C8—C12—O2	−0.5 (2)
C2—C3—C4—C5	0.3 (3)	C9—C8—C12—C11	−0.6 (2)
C3—C4—C5—C6	1.2 (2)	C5—C8—C12—C11	178.64 (13)
C3—C4—C5—C8	−175.40 (14)	C10—C11—C12—O2	179.37 (14)
C4—C5—C6—C7	−1.5 (2)	C16—C11—C12—O2	0.6 (2)
C8—C5—C6—C7	175.13 (14)	C10—C11—C12—C8	0.2 (2)
C5—C6—C7—C2	0.1 (2)	C16—C11—C12—C8	−178.60 (14)
O1—C2—C7—C6	−178.32 (13)	O3—C10—C13—C14	−177.57 (13)
C3—C2—C7—C6	1.5 (3)	C11—C10—C13—C14	1.2 (2)
C4—C5—C8—C9	39.5 (2)	C17—O4—C14—C13	−7.9 (2)
C6—C5—C8—C9	−136.98 (16)	C17—O4—C14—C15	170.69 (14)
C4—C5—C8—C12	−139.76 (16)	C10—C13—C14—O4	176.99 (14)
C6—C5—C8—C12	43.8 (2)	C10—C13—C14—C15	−1.6 (2)
C12—C8—C9—O3	0.8 (2)	O4—C14—C15—C16	−178.21 (13)
C5—C8—C9—O3	−178.50 (13)	C13—C14—C15—C16	0.5 (2)
C10—O3—C9—C8	−0.5 (2)	C14—C15—C16—C11	1.1 (2)
C9—O3—C10—C13	178.77 (13)	C10—C11—C16—C15	−1.5 (2)
C9—O3—C10—C11	0.0 (2)	C12—C11—C16—C15	177.38 (14)
O3—C10—C11—C16	179.00 (13)	C14—O4—C17—C18	−170.80 (14)
C13—C10—C11—C16	0.3 (2)		

### Hydrogen-bond geometry (Å, °)

**Table:**

*D*—H···*A*	*D*—H	H···*A*	*D*···*A*	*D*—H···*A*
C9—H9···O2^i^	0.93	2.52	3.105 (2)	121
C16—H16···O3^ii^	0.93	2.60	3.315 (2)	134
C17—H17B···O2^iii^	0.97	2.58	3.358 (3)	138

Symmetry codes: (i) *x*+1, *y*, *z*; (ii) *x*−1, *y*, *z*; (iii) −*x*, −*y*+2, −*z*.
